# Association Between Pulmonary Artery Diameter on CT and Clinical Outcomes in COVID‐19: A 1000‐Patient Retrospective Analysis

**DOI:** 10.1155/rrp/8305125

**Published:** 2026-11-15

**Authors:** Fatemeh Yarmahmoodi, Maryam Elahi, Mahdi Saeedi-Moghadam, Seyed Mostajab Razavinejad, Banafsheh Zeinali-Rafsanjani

**Affiliations:** ^1^ Medical Imaging Research Center Shiraz University of Medical Sciences Shiraz Iran sums.ac.ir; ^2^ Department of Radiology Shiraz University of Medical Sciences Shiraz Iran sums.ac.ir; ^3^ Neonatal Research Center Shiraz University of Medical Sciences Shiraz Iran sums.ac.ir

**Keywords:** chest CT, COVID-19, lung involvement, mortality, pulmonary artery diameter

## Abstract

**Background:**

We evaluated the association between main pulmonary artery (PA) diameter on chest CT and CT‐defined lung involvement, intensive care unit (ICU) hospitalization, and in‐hospital mortality in patients hospitalized with COVID‐19.

**Methods:**

This retrospective cohort included 1000 adults with PCR‐confirmed COVID‐19 treated at Namazi and Shahid Faghihi Hospitals in 2020. For each patient, the first routine noncontrast chest CT obtained after admission was analyzed, provided it had been performed within the first 72 h. Main PA diameter was measured manually on axial images proximal to the PA bifurcation. Associations with age, sex, CT severity, ICU hospitalization, and in‐hospital outcome were evaluated.

**Results:**

The mean age was 56 ± 17.7 years; 525 patients (52.5%) were men and 475 (47.5%) were women. Mean PA diameter was 27.07 ± 3.84 mm. Men had a larger mean PA diameter than women (27.40 ± 3.77 vs. 26.69 ± 3.88 mm; *p* = 0.004), and age showed a weak positive correlation with PA diameter (*r* = 0.282, *p* < 0.001). PA diameter increased across CT severity categories and was greater among patients who died and among those hospitalized in the ICU (all *p* < 0.001).

**Conclusions:**

Larger PA diameter was associated with greater CT‐defined lung involvement, ICU hospitalization, and in‐hospital mortality. Because the analyses were unadjusted and observational, these findings do not establish independent or incremental prognostic value.

## 1. Introduction

The COVID‐19 pandemic, emerging in late 2019, has had profound global impacts, disrupting healthcare systems, economies, and societal stability. [1–3]. The reference diagnostic tool for diagnosing this disease is real‐time polymerase chain reaction or PCR [4]. Computed tomography (CT) can provide complementary information about pulmonary involvement in the clinical evaluation of COVID‐19 [5–7]. Moreover, lung CT can be helpful in monitoring and determining prognosis in patients with COVID‐19 [6, 8].

Some studies pointed to vascular changes in COVID‐19 chest CT [9–11]. Autopsy studies also showed interstitial pneumonia with alveolar damage, pulmonary arteriole thrombosis, and right ventricular dilatation [12, 13]. These findings indicate that the increase in the afterload of the right ventricle due to endothelial damage accompanied by microthrombosis of the pulmonary vessels could be one of the main pathological factors causing critical conditions in COVID‐19 patients [14, 15]. Two echocardiography studies in patients with COVID‐19 showed larger heart cavities, decreased right ventricular function, and increased pulmonary artery (PA) systolic pressure in deceased individuals [16, 17]. These observations provide a rationale for evaluating main PA diameter as a marker of pulmonary vascular involvement. An increase in the main pulmonary artery diameter (MPAD) indicates hemodynamic instability due to acute central pulmonary embolism and heart failure [18–20]. Also, measuring the maximum diameter of the PA and the ascending aorta (Ao) as well as the ratio of these two (PA‐Ao ratio) in lung CT without the use of contrast media is a noninvasive option in the diagnosis and screening of pulmonary hypertension [20–24]. Likewise, several studies show the relationship of these indicators (PA, PA‐Ao ratio) with the disease severity and treatment outcome in patients with COVID‐19 [9, 10, 25–29].

Although pulmonary vascular abnormalities have been reported in COVID‐19, evidence on the relationship between MPAD and clinical outcome has remained limited, particularly in Iranian populations. We therefore examined the association of PA diameter on admission chest CT with CT‐defined lung involvement and in‐hospital outcomes.

## 2. Materials and Methods

We performed a retrospective cohort analysis to assess the association between MPAD and CT‐defined lung involvement and in‐hospital outcomes in patients with COVID‐19. This analysis used the same underlying 1000‐patient cohort previously described in [30]; the present study addressed a distinct imaging endpoint focused on PA diameter. Briefly, 1000 PCR‐confirmed COVID‐19 patients admitted to Namazi and Shahid Faghihi Hospitals in 2020 were analyzed using the first routine noncontrast chest CT obtained within 72 h of admission. Eligible patients were older than 18 years. The patients with a history of pneumonia for reasons other than COVID‐19, a history of pulmonary hypertension, a history of pulmonary thromboembolism, a history of severe COPD and interstitial lung diseases, or patients with incomplete medical documents were excluded from the study. Exclusion criteria were determined through review of the patients’ documented medical history. No pre‐COVID chest CT examination was available in the institutional PACS for the included patients; therefore, baseline PA diameter before SARS‐CoV‐2 infection could not be assessed.

The first chest CT examination obtained after hospital admission was included in the analysis. All examinations were routine noncontrast chest CT studies performed within 72 h of admission for evaluation of COVID‐19 pneumonia; intravenous iodinated contrast material was not administered, and the examinations were not performed as CT pulmonary angiography. Scans were obtained at Namazi and Shahid Faghihi Hospitals using Philips Ingenuity and Philips Brilliance 16 CT systems. Acquisition settings varied slightly across examinations; representative settings were approximately 120 kVp, 30 mA, and 0.63‐mm slice thickness for the Philips Ingenuity scanner and 90 kVp, 20 mA, and 0.75‐mm slice thickness for the Philips Brilliance 16 scanner. Because these were noncontrast routine chest CT examinations rather than CTPA, acute pulmonary embolism was not systematically evaluated on the study scans.

The main PA trunk diameter was measured manually on axial CT images proximal to the PA bifurcation, with the caliper placed across the vessel diameter. PA diameter was analyzed as a continuous variable, and no prespecified dichotomous threshold was used to define PA enlargement. Measurements were performed by a radiology resident after standardized instruction from an attending radiologist with more than 10 years of experience. A single reader performed all measurements; therefore, interobserver agreement was not assessed, and a formal intraobserver repeatability study was not performed. Formal blinding to clinical outcomes was not incorporated into the retrospective image‐review workflow.

The data were collected using the checklist prepared from the patient’s document and the CT findings recorded in the PACS. This checklist had three parts. The first part included the patient’s demographic information, such as age and sex. The second part included information related to the inpatient department, underlying disease history, clinical information, and chest CT findings of the patients, including the severity of the involvement, the location and the type of involvement, and PA size measurement. The third part was data related to the death and recovery of patients. The study checklist categorized CT lung involvement by percentage as mild (0%–25%), moderate (26%–49%), severe (50%–75%), or very severe (> 75%). ICU hospitalization during the index admission was recorded as an in‐hospital clinical outcome.

The original analyses were performed using SPSS Version 25. Normality was assessed using the Kolmogorov–Smirnov test. Continuous variables were summarized as means and standard deviations, and categorical variables as counts and percentages. Independent‐samples *t*‐tests were used for two‐group comparisons, and Pearson’s correlation coefficient was used to examine the relationship between age and PA diameter. During revision, the comparison across the four CT severity categories was reconstructed from the reported severity‐group counts, means, and standard deviations using Welch’s one‐way analysis of variance and Games–Howell pairwise comparisons. This was an aggregate‐data reanalysis; the individual‐level analytic dataset was not rerun. Games–Howell adjustment applied to the six pairwise severity comparisons, rather than to all tests in the study. The significance level was 0.05. The reported associations were unadjusted; no multivariable regression model was performed. Age‐group means were treated descriptively; the inferential association between continuous age and PA diameter was assessed with Pearson’s correlation coefficient.

## 3. Results

The mean age was 56 ± 17.7 years, and 52.5% of patients were men. CT‐defined lung involvement was mild in 483 patients (48.3%), moderate in 284 (28.4%), severe in 140 (14.0%), and very severe in 93 (9.3%).

84.1% of patients had recovered, and 15.9% had died. One hundred and sixty‐two patients (16.2%) were admitted to the ICU. Among these, 63 (38.9%) had recovered and 99 (61.1%) had died. Mean PA diameter was 27.07 ± 3.84 mm (range, 19–43 mm). Men had a larger mean PA diameter than women (27.40 ± 3.77 mm vs. 26.69 ± 3.88 mm; *p* = 0.004). PA diameter increased descriptively across age groups, with the highest means in the 60–70 and ≥ 70‐year groups and the lowest mean in patients younger than 30 years (Table 1).

**TABLE 1:** Pulmonary artery diameter according to age group and sex.

Variable	Category	Mean PA diameter (mm)	SD	*p*
Sex	Male (*n* = 525)	27.40	3.77	0.004
	Female (*n* = 475)	26.69	3.88	

Age	< 30 (*n* = 68)	25.07	3.03	—
	30–40 (*n* = 146)	25.64	3.11	
	40–50 (*n* = 160)	26.18	3.18	
	50–60 (*n* = 159)	26.83	3.63	
	60–70 (*n* = 225)	27.85	3.97	
	≥ 70 (*n* = 242)	28.50	4.15	

*Note:* Age‐group values are presented descriptively. The association between continuous age and PA diameter was tested using Pearson correlation (*r* = 0.282, *p* < 0.001).

Figure 1 shows the relationship between patient age and PA diameter. Pearson correlation analysis demonstrated a weak positive association between age and PA diameter (*r* = 0.282, *p* < 0.001). Although statistically significant, the magnitude of the correlation was modest.

**FIGURE 1:**
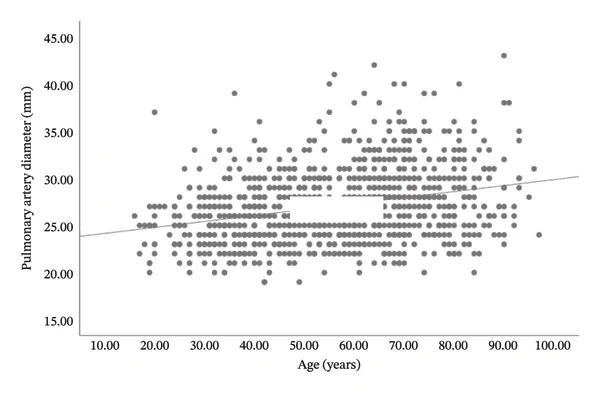
Relationship between patient age and pulmonary artery diameter (Pearson *r* = 0.282, *p* < 0.001).

Table 2 summarizes PA diameter according to CT severity, in‐hospital outcome, and ICU hospitalization. Mean PA diameter was highest in the very severe group and lowest in the mild group. The overall difference across the four severity groups remained statistically significant on Welch’s ANOVA (F(3, 292.84) = 25.55, *p* < 0.001). Games–Howell post hoc comparisons showed that all six pairwise group differences remained significant after adjustment; the largest adjusted *p* value was 0.039 for the moderate‐versus‐severe comparison (all other adjusted *p* values ≤ 0.007). Mean PA diameter was significantly greater in patients who died than in those who recovered and in patients hospitalized in the ICU than in those who were not. The revision‐stage Welch and Games–Howell calculations were reconstructed from reported aggregate group summaries rather than from individual‐level patient records.

**TABLE 2:** Pulmonary artery diameter according to CT severity, in‐hospital outcome, and ICU hospitalization.

Variable	Category	Mean PA diameter (mm)	SD	*p*
Severity of involvement	Mild (*n* = 483)	26.15	3.72	< 0.001
	Moderate (*n* = 284)	27.10	3.51	
	Severe (*n* = 140)	28.10	3.66	
	Very severe (*n* = 93)	29.96	4.57	

Outcome	Recovery (*n* = 841)	26.59	3.62	< 0.001
	Death (*n* = 159)	29.56	4	

ICU hospitalization	Yes (*n* = 162)	28.74	4.01	< 0.001
	No (*n* = 838)	26.75	3.72	

*Note:* Group sizes were 483 mild, 284 moderate, 140 severe, and 93 very severe; 841 patients recovered and 159 died; 162 were hospitalized in the ICU and 838 were not.

## 4. Discussion

Prior to the outbreak of COVID‐19, studies had shown that PA enlargement on chest CT was associated with pulmonary hypertension in other clinical settings [21, 22, 31], reflecting the severity [31] and duration of the disease [32]. Pulmonary hemodynamic impairment in severe COVID‐19 may accompany extensive parenchymal consolidation, inflammatory vascular changes, and thromboembolic phenomena [32, 33]. CT measurement of PA diameter is a noninvasive imaging marker used in the assessment of pulmonary hypertension. In the present study, mean PA diameter was higher in patients with more severe CT findings and was associated with in‐hospital mortality. These observations are consistent with pulmonary vascular involvement in severe COVID‐19 but do not establish causality.

Our prior publication used the same cohort to examine thyroid involvement [30]; the present analysis showed an association between larger PA diameter and poorer in‐hospital outcomes. Because these associations were not adjusted for other clinical or imaging markers of disease severity, they should not be interpreted as demonstrating an independent effect of PA size.

Chest CT findings, particularly the extent of lung involvement, play a pivotal role in guiding clinical management and risk stratification for COVID‐19 patients. In our cohort, CT‐defined lung involvement was mild in 48.3% of patients, moderate in 28.4%, severe in 14.0%, and very severe in 9.3%.

Similar findings were observed by Hafez and Francone et al. [34, 35], in which the disease severity was classified as mild, moderate, or severe using lung opacity and CT‐SS calculation [36]. Their results showed that most patients had moderate (52.5%) and severe (27.5%) CT‐SS. According to recent studies, chest CT scan plays an essential role in the early diagnosis of COVID‐19 and in evaluating the severity of pulmonary involvement [37, 38]. In addition, it can provide prognostic information related to progression to severe stage [39]. It is also considered a valuable tool to identify patients who need invasive treatments instead of routine treatment [40]. So far, the level of involvement and the rapid progress of CT findings are the most critical clinical criteria for COVID severity [41].

In severe COVID‐19, inflammatory and endothelial injury may affect the pulmonary circulation. In the present analysis, larger PA diameter was associated with greater CT‐defined disease severity and adverse in‐hospital outcomes. Inflammatory cytokines, including IL‐6, IL‐10, and tumor necrosis factor α, have been implicated in acute respiratory distress syndrome and pulmonary vascular dysfunction in SARS‐CoV‐2 infection [42, 43]. PA diameter can be measured rapidly on routine CT without dedicated software and may provide a simple quantitative vascular marker. In prior COVID‐19 cohorts, enlarged MPAD has also been associated with adverse outcome [25]. In our cohort, mean PA diameter was 27.07 ± 3.84 mm (range, 19–43 mm) and increased across CT severity categories.

For context, the Framingham Heart Study reported sex‐specific upper reference values of 27 mm for women and 29 mm for men [44]. In Spagnolo et al.’s study [10], the median PA diameter was 31 mm (IQR, 28–33 mm). Spagnolo et al. also reported that the PA/A ratio was associated with the extent of COVID‐19 pneumonia [10]. The study of Riachy et al. [45] reported an association between PA enlargement and mortality. In the study of Yildiz et al. [26], patients with severe CT findings of COVID‐19 pneumonia had a larger MPAD. In the study of Eslami et al. [9], greater lung involvement was associated with higher PA/A values. In the study of Aoki et al. [27], enlargement of pulmonary vessels (maximum subsegmental diameter > 4 mm) was observed in 66 patients (75.9%) and in all patients with severe or critical disease. Multivariate logistic analysis by Aoki et al. [27] showed that subsegmental vessel diameter was an independent predictor of disease severity (odds ratio 3.05, *p* = 0.018). These findings support subsegmental vascular enlargement as a relevant CT feature of acute COVID‐19.

In the present study, PA diameter was analyzed as a continuous variable rather than dichotomized using a single enlargement threshold. The sex‐specific Framingham values cited above (approximately 29 mm in men and 27 mm in women) are therefore presented as contextual reference limits rather than as a study cutoff. Age and sex were examined in relation to PA diameter, whereas body‐size measurements were not available. Ascending aortic diameter was also not recorded, so the PA‐to‐ascending‐aorta ratio could not be calculated in this cohort, although this ratio may provide complementary information when interpreting PA size.

A dilated PA trunk may reflect several processes in severe COVID‐19. Proinflammatory mediators can increase pulmonary vascular resistance and right ventricular afterload [46], while hypoxemia and thrombotic complications may further affect the pulmonary circulation [47]. In Zhu et al.’s cohort, patients with a PA trunk diameter > 29 mm had higher frequencies of pleural and pericardial effusion and several comorbidities than those with smaller PA diameters [28]. These observations support PA diameter as a marker associated with more severe systemic and cardiopulmonary involvement, but they do not establish a specific mechanism in our cohort.

In the present study, mean PA diameter was higher among patients who died and among those admitted to the ICU. This pattern may reflect the pulmonary vascular and hemodynamic consequences that accompany more severe COVID‐19, including hypoxemia and increased pulmonary vascular resistance. However, the observational design and unadjusted analyses do not allow a causal interpretation or establish that PA diameter provides prognostic information independent of overall disease severity. PA measurement may therefore be useful as an adjunctive imaging marker for risk stratification, but its clinical utility should be confirmed in multivariable and prospective studies.

These findings are broadly consistent with prior studies. Zhu et al. [28] reported that a PA trunk diameter > 29 mm at admission was independently associated with in‐hospital mortality in 180 patients with COVID‐19. Esposito et al. [25] similarly found that moderate‐to‐severe PA enlargement (MPAD ≥ 31 mm) on CT performed within 72 h of admission was independently associated with mortality. Erdoğan et al. [29] also reported that larger MPA diameter was independently associated with in‐hospital mortality in a cohort of 255 patients with severe or critical COVID‐19.

Riachy et al. [45] likewise reported an association between PA enlargement and mortality in hospitalized patients with COVID‐19, although the diagnostic performance of PA diameter varied across studies. Eslami et al. [9] found that more extensive lung involvement was associated with higher PA/A values. Outside COVID‐19, enlargement of the main PA on CT is also recognized as an imaging finding associated with pulmonary hypertension [31, 32]. Together, these observations support PA diameter as a clinically relevant imaging marker, while the present unadjusted analysis cannot determine whether it provides prognostic information independent of established clinical and radiological factors.

Longitudinal evidence suggests that PA enlargement may be at least partly reversible after recovery. Matthaiou et al. reported a significant decrease in mean MPAD from 31 ± 3 mm during acute COVID‐19 pneumonia to 28 ± 3 mm on follow‐up CT performed 2–18 months later [48]. This finding supports the possibility that PA dilatation reflects, at least in part, a dynamic vascular response to acute disease rather than a fixed abnormality. Because follow‐up CT examinations were not systematically collected in our cohort, we could not assess whether PA diameter regressed after recovery or whether longitudinal changes were associated with clinical outcomes.

Whether PA enlargement represents a distinct manifestation of COVID‐19‐related pulmonary vascular injury or primarily tracks the overall severity of acute illness cannot be resolved from the present data. Because no multivariable or incremental‐value analysis was performed, we cannot determine whether PA diameter adds prognostic information beyond age, sex, CT severity, cardiovascular comorbidities, or other established clinical markers. Future studies should compare models with and without PA diameter and assess calibration as well as discrimination before routine prognostic use is considered.

This study has several limitations. Its retrospective design and recruitment from two university‐affiliated hospitals in the same city may introduce selection bias. The interval from symptom onset to hospital admission was not consistently available, and body mass index was not recorded. No pre‐COVID chest CT was available for comparison, so pre‐existing PA dilatation or interval change from baseline could not be assessed. Although patients with documented pulmonary hypertension, prior pulmonary thromboembolism, severe COPD, and interstitial lung disease were excluded by chart review, unrecognized pre‐existing causes of PA enlargement cannot be fully excluded. Follow‐up CT examinations were not systematically collected. PA measurements were obtained by a single reader without formal blinding, and interobserver and intraobserver reproducibility were not assessed. The analyses were unadjusted for cardiovascular comorbidities and other established markers of COVID‐19 severity, leaving potential residual confounding. Because the admission examinations were noncontrast chest CT rather than CTPA, acute pulmonary embolism could not be systematically excluded. The original individual‐level analytic dataset was not available during revision, preventing patient‐level reanalysis and reconciliation of a small inconsistency between the pooled severity‐group summary statistics and the overall reported PA mean/SD. Accordingly, the revision‐stage Welch/Games–Howell results should be interpreted as aggregate‐data reconstructions. The available study documentation did not provide a more detailed description of how lobe‐level severity assessments were combined into a patient‐level category, and the exact timing of ICU transfer relative to the index CT was not available.

## 5. Conclusion

Larger PA diameter was associated with greater CT‐defined lung involvement, ICU admission, and in‐hospital mortality in this retrospective cohort. These findings support PA diameter as a potentially useful imaging marker of disease severity and adverse outcome. Because the analyses were unadjusted and the study was observational, however, independent or incremental prognostic value cannot be inferred and should be evaluated in prospective multivariable studies.

**Nomenclature**
ICU: Intensive care unit
CT: Computed tomography
PA: Pulmonary artery
Ao: Ascending aorta
PCR: *Polymerase chain reaction*
PACS: Picture Archiving and Communication System
COPD: Chronic obstructive pulmonary disease

## Author Contributions

Study conception and design: Fatemeh Yarmahmoodi, Banafsheh Zeinali‐Rafsanjani, and Maryam Elahi.

Data acquisition: Fatemeh Yarmahmoodi, Mahdi Saeedi‐Moghadam, and Seyed Mostajab Razavinejad.

Analysis and interpretation: Fatemeh Yarmahmoodi and Banafsheh Zeinali‐Rafsanjani.

Statistical analysis: Banafsheh Zeinali‐Rafsanjani.

Drafting of the manuscript: Fatemeh Yarmahmoodi, Mahdi Saeedi‐Moghadam, and Seyed Mostajab Razavinejad.

Critical revision for important intellectual content: Maryam Elahi.

Administrative, technical, and material support: Banafsheh Zeinali‐Rafsanjani and Seyed Mostajab Razavinejad.

## Funding

This work was supported by the Vice‐Chancellery for Research, School of Medicine, Shiraz University of Medical Sciences, under research project no. 24191.

## Ethics Statement

This study was approved by the Research Ethics Committee of the School of Medicine, Shiraz University of Medical Sciences (IR.SUMS.MED.REC.1400.464; approval date: November 27, 2021) and was conducted in accordance with the Declaration of Helsinki.

## Consent

The authors have nothing to report.

## Conflicts of Interest

The authors declare no conflicts of interest.

## Data Availability

The original individual‐level analytic dataset is no longer available to the authors in a form suitable for independent reanalysis. Source medical records and CT images remain subject to institutional governance and patient‐confidentiality requirements.
